# Pulsed dye versus neodymium-doped yttrium aluminum garnet lasers for refractory warts: a systematic review and meta-analysis

**DOI:** 10.12701/jyms.2026.43.6

**Published:** 2025-12-22

**Authors:** Joon-Goon Kim

**Affiliations:** Department of Dermatology, Yeungnam University College of Medicine, Daegu, Korea

**Keywords:** Dyes lasers, Meta-analysis, Solid-state lasers, Systematic review, Treatment failure, Warts

## Abstract

**Background:**

Refractory cutaneous warts often persist despite conventional treatments. Pulsed dye lasers (PDLs) and long-pulsed neodymium-doped yttrium aluminum garnet (Nd:YAG) lasers are commonly used in clinical practice. However, comparative data on refractory cases remain limited.

**Methods:**

Following PRISMA 2020 guidelines, PubMed, Embase, Scopus, and Cochrane Library were searched through October 11, 2025, for studies evaluating PDL or Nd:YAG treatment for refractory warts. Head‑to‑head studies were meta-analyzed using Inverse Variance fixed‑effect odds ratios (ORs), whereas single‑arm evidence was summarized narratively following the SWiM guidelines. The primary outcome was complete clearance, with hemorrhagic complications as the key safety outcome.

**Results:**

Fifteen studies met the inclusion criteria: three head‑to‑head (n=224; 115 PDLs and 109 Nd:YAG lasers) and 12 single‑arm series. Complete clearance was similar between PDLs and Nd:YAG lasers (OR, 1.29; 95% confidence interval [CI], 0.66–2.50; I²=0%). Hemorrhagic complications occurred less frequently with PDLs (OR, 0.23; 95% CI, 0.07–0.74; I²=22%). Single-arm studies indicated broad clearance ranges (PDL, 5.1%–89.0%; Nd:YAG, 9.1%–100%).

**Conclusion:**

PDLs and Nd:YAG lasers demonstrated comparable efficacies in treating refractory warts, and PDLs conferred a safety advantage for hemorrhagic events. The lesion site, thickness, bleeding tolerance, and patient context should be considered when selecting the treatment modality.

## Introduction

Cutaneous warts (verrucae) are benign epithelial proliferations caused by infection of keratinocytes with human papillomavirus (HPV), with an estimated prevalence of 5% to 30% in children and young adults [1,2]. Common warts account for approximately 70% of cases, whereas plantar and plane warts account for 24% and 3.5%, respectively [3]. Although many warts undergo spontaneous resolution within 1 to 2 years in children, clearance in adults can be significantly slower, and persistence for 5 to 10 years is common. Traditional first-line treatments, including salicylic acid treatment and cryotherapy, frequently fail to achieve complete remission, particularly in recalcitrant cases [1].

Refractory or recalcitrant warts are defined as lesions that persist despite multiple attempts at conventional treatment, presenting a significant therapeutic challenge in dermatological practice. These resistant warts are often associated with specific HPV genotypes, particularly HPV types 2, 27, and 57, and tend to occur at anatomically challenging sites, such as periungual and plantar locations [2]. Therefore, the management of refractory warts requires alternative treatment modalities beyond the standard approaches.

Over the past few decades, laser therapy has emerged as a promising treatment option for recalcitrant warts. Among the various laser modalities, non-ablative lasers, including pulsed dye lasers (PDLs) and neodymium-doped yttrium aluminum garnet (Nd:YAG) lasers, have demonstrated favorable efficacy in multiple studies. PDLs operate at wavelengths of 585 to 595 nm, targeting hemoglobin within dilated capillaries that supply warts, thereby inducing ischemic necrosis. Nd:YAG lasers, operating at a wavelength of 1,064 nm, offer deeper tissue penetration and similar vascular-targeting mechanisms, targeting hemoglobin to coagulate and destroy dermal blood vessels in warts. This longer wavelength allows deeper penetration into the hyperkeratotic epidermis than PDLs. Histologically, it causes dermo-epidermal junction separation, epidermal necrosis, and capillary coagulation. A systematic review of 35 studies involving 2,149 patients reported clearance rates ranging from 47% to 100% for PDL and 46% to 100% for Nd:YAG laser treatment of nongenital warts [3].

Despite growing evidence supporting the efficacy of both PDLs and Nd:YAG lasers in wart treatment, direct comparative studies between these two non-ablative modalities remain limited, particularly in the context of refractory warts. Therefore, this systematic review was conducted with a particular focus on refractory cutaneous warts to (1) synthesize head‑to‑head comparisons of PDL versus Nd:YAG laser therapy regarding complete clearance and key safety outcomes, and (2) contextualize the performance of each modality from single‑arm studies without making indirect between‑laser comparisons. This approach aims to align clinical decision-making with the best available comparative evidence while transparently summarizing single-arm data for real-world applicability.

## Methods

**Ethics statement:** Not applicable.

### 1. Data sources and search strategy

This systematic review followed the Preferred Reporting Items for Systematic Reviews and Meta-Analyses (PRISMA) 2020 guidelines (the protocol was prospectively designed and not registered). The narrative syntheses of single-arm studies adhered to the Synthesis Without Meta-analysis (SWiM) guidelines. PubMed, Embase, Scopus, and the Cochrane Library were searched on October 11, 2025, using the following terms: (wart OR verruca) AND (dye OR PDL OR neodymium‑doped yttrium‑aluminum‑garnet OR YAG) AND (recalcitrant OR resistant OR refractory OR persistent OR recurrent OR relapse). No restrictions were imposed on the dates. Full non-English texts were excluded from eligibility.

### 2. Study selection

Studies were included if they met the following criteria: (1) patients with refractory, recalcitrant, or treatment-resistant warts (defined as failure of at least one prior conventional therapy); (2) treatment with a PDL (585–595 nm) or Nd:YAG laser (1,064 nm); (3) reporting of complete clearance rates; and (4) prospective or retrospective cohort studies, comparative trials, or case series with ≥10 patients. The exclusion criteria were as follows: (1) case reports with <10 patients focusing solely on technique description, (2) studies combining laser with concurrent adjuvant therapies, (3) studies without a clear definition of refractory status, (4) duplicate publications, (5) conference abstracts without full text, and (6) studies not published in English.

The study selection was performed in two phases. First, titles and abstracts were screened against the eligibility criteria. Subsequently, the full texts of potentially eligible studies were retrieved and assessed for final inclusion. Studies were categorized as: (1) head-to-head comparative studies eligible for meta-analysis or (2) single-arm studies eligible for descriptive synthesis only. Any uncertainty regarding eligibility or categorization was resolved through reexamination of the original inclusion criteria and consultation with a senior dermatologist when necessary. The study selection process was documented using a PRISMA flow diagram.

### 3. Data extraction and quality assessment

Data were extracted using standardized forms to capture study characteristics (design, setting, and sample size), patient numbers, wart characteristics (location and type), laser parameters (wavelength, fluence, spot size, pulse duration, and number of sessions for clearance), and outcomes (complete clearance, defined as 100% lesion resolution, and adverse events).

**Fig. 1.**
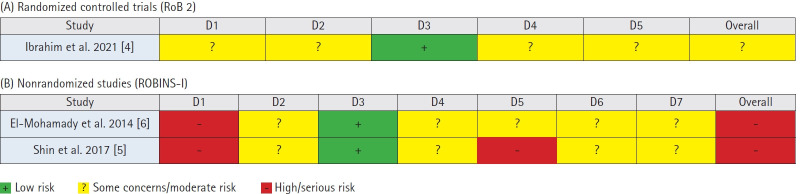
Traffic-light plot of included studies. (A) Randomized controlled trials (RoB 2), (B) nonrandomized studies (ROBINS-I). For randomized controlled trials, the RoB 2 domains included the randomization process (D1), deviations from intended interventions (D2), missing outcome data (D3), measurement of the outcome (D4), and selection of the reported result (D5). For nonrandomized studies, the ROBINS-I domains comprised confounding (D1), selection of participants (D2), classification of interventions (D3), deviations from intended interventions (D4), missing data (D5), measurement of outcomes (D6), and selection of the reported result (D7).

The risk of bias was assessed using a qualitative domain-based approach. For head-to-head comparative studies, RoB 2 (for randomized studies) and ROBINS‑I (for nonrandomized studies) tools were used, and results were summarized descriptively (traffic‑light plot, Fig. 1). For single-arm studies, descriptive quality assessment focused on selection, performance, detection, attrition, and reporting biases. Study quality was rated on a 5-point scale based on methodological rigor.

### 4. Data synthesis and analysis

#### 1) Meta-analysis of head-to-head comparative studies

A quantitative meta-analysis was conducted on the three head-to-head comparative studies that directly compared PDL with Nd:YAG laser treatments. Odds ratios (ORs) with corresponding 95% confidence intervals (CIs) were calculated as primary effect measures for efficacy and safety outcomes. ORs were selected because they are appropriate for binary outcomes and allow for a clear interpretation of relative treatment effects.

Fixed-effects models based on the Inverse Variance method were used for all meta-analyses.

#### 2) Assessment of statistical heterogeneity

Statistical heterogeneity across head-to-head comparative studies was assessed using the I² statistic, with values interpreted according to standard thresholds: I²<25%, low heterogeneity; 25%≤I²≤75%, moderate heterogeneity; and I²>75%, high heterogeneity.

#### 3) Descriptive synthesis of single-arm studies

Single-arm studies (i.e., those evaluating PDL monotherapy or Nd:YAG monotherapy without direct comparison groups) were qualitatively and descriptively summarized. For these studies, the range of complete clearance rates observed across the PDL and Nd:YAG studies was calculated and reported separately.

Quantitative pooling or statistical comparisons of single-arm PDL and single-arm Nd:YAG studies were not performed because of the high risk of confounding inherent in such indirect comparisons. Differences in patient populations, wart characteristics, treatment protocols, outcome assessment methods, and follow-up durations across single-arm studies precluded valid statistical inferences regarding comparative efficacy.

The single-arm studies were included in the systematic review to provide contextual evidence for each laser modality and to describe the range of treatment responses reported in the literature. However, conclusions regarding comparative efficacy and safety were derived exclusively from the head-to-head comparative studies included in the meta-analysis.

Publication bias was not assessed using funnel plots because the number of eligible studies was insufficient (<10). All analyses were conducted using RevMan 5.4 software (Cochrane Collaboration, Copenhagen, Denmark). Statistical significance was set at *p*<0.05.

## Results

### 1. Literature search and selection of studies

**Fig. 2.**
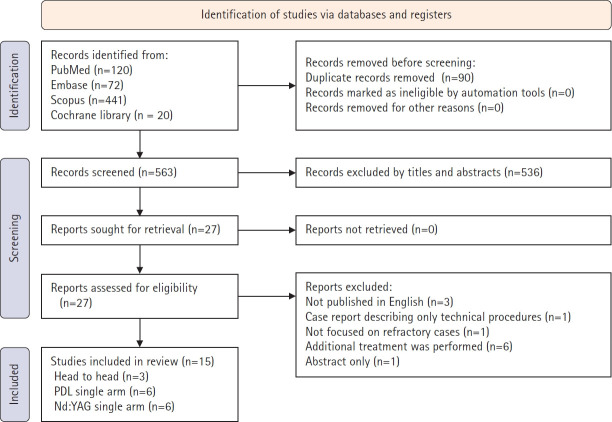
PRISMA (Preferred Reporting Items for Systematic Reviews and Meta-Analyses) 2020 flow diagram. PDL, pulsed dye laser; Nd:YAG, neodymium-doped yttrium aluminum garnet.

The database search identified 653 records (PubMed, 120; Embase, 72; Scopus, 441; and Cochrane Library, 20). After removing 90 duplicate articles, 563 titles and abstracts were screened, of which 536 were excluded. Twenty-seven full texts were assessed for eligibility, and all were retrieved. Twelve reports were excluded for predefined reasons (non‑English, case reports describing only technical procedures, absence of refractory cases, concurrent additional treatments, or abstract‑only reports). Ultimately, 15 studies were included in the qualitative synthesis: three head‑to‑head comparisons of PDLs and Nd:YAG lasers, six single‑arm PDL studies, and six single‑arm Nd:YAG laser studies (Fig. 2).

### 2. Study characteristics and quality

Across all included reports, participants had refractory (recalcitrant/resistant) warts following the failure of at least one prior therapy. Interventions employed a PDL (585–595 nm) or Nd:YAG laser (1,064 nm) according to each study protocol. The study designs were predominantly nonrandomized prospective or retrospective cohorts. Outcome definitions were consistent, with complete clearance defined as 100% lesion resolution.

### 3. Comparative efficacy of pulsed dye lasers versus Nd:YAG lasers (complete clearance)

**Fig. 3.**
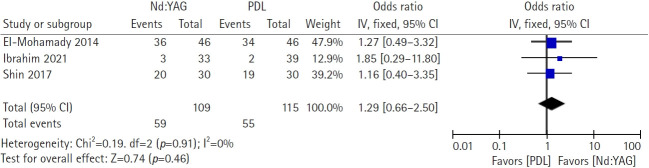
Treatment efficacy between pulsed dye lasers (PDLs) and neodymium-doped yttrium aluminum garnet (Nd:YAG) lasers for refractory warts. CI, confidence interval; df, degree of freedom; IV, inverse variance.

Three head‑to‑head studies (Ibrahim et al. [4] in 2021, Shin et al. [5] in 2017, and El‑Mohamady et al. [6] in 2014) comprising 224 participants were pooled using a fixed‑effect model (Nd:YAG, n=109; PDL, n=115). Complete clearance occurred in 59 of 109 participants in the Nd:YAG arms and 55 of 115 in the PDL arms. The meta‑analysis revealed no significant difference between PDL and Nd:YAG in complete clearance (OR, 1.29; 95% CI, 0.66–2.50; Z=0.74; *p*=0.46). Statistical heterogeneity was negligible (*χ*^2^=0.19, degrees of freedom [df]=2, *p*=0.91, I²=0%), supporting the use of a fixed‑effect model. These findings indicated a broadly comparable efficacy for complete wart clearance between the two laser modalities in direct comparisons (Fig. 3).

### 4. Adverse events (hematoma/hemorrhagic bullae)

**Fig. 4.**
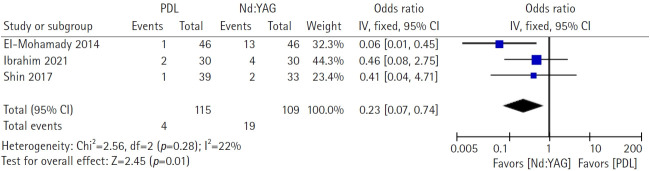
Hematoma/hemorrhagic bullae adverse effect between pulsed dye lasers (PDLs) and neodymium-doped yttrium aluminum garnet (Nd:YAG) lasers for refractory warts. CI, confidence interval; df, degree of freedom; IV, inverse variance

Hemorrhagic complications were analyzed across the same three comparative studies. These events were less frequent with PDLs (4/115) than with Nd:YAG lasers (19/109). Pooled analysis using a fixed‑effect model demonstrated a statistically significant advantage for PDLs (OR, 0.23; 95% CI, 0.07–0.74; Z=2.45; *p*=0.01). Heterogeneity was low to moderate (*χ*^2^=2.56, df=2, *p*=0.28, I²=22%). Thus, although the clearance efficacy was similar, PDL use was associated with a significantly lower risk of hemorrhagic adverse events than Nd:YAG laser use in head-to-head studies (Fig. 4).

### 5. Pulsed dye laser single-arm outcomes

Six single-arm PDL studies indicated complete clearance rates ranging from 5.1% to 89.0%. Given the between-study variability in patient selection, lesion characteristics, and treatment parameters, pooled estimates were not generated. The outcomes are summarized in Table 1. Collectively, these series confirmed that PDLs can achieve complete clearance in a meaningful proportion of recalcitrant cases, albeit with substantial variability across settings. When adverse events were reported, hemorrhagic complications were uncommon, which was consistent with the comparative safety analysis. Detailed study‑level characteristics and outcome proportions are summarized in Table 1.

### 6. Nd:YAG laser single-arm outcomes

Six single-arm Nd:YAG studies indicated complete clearance rates ranging from 9.1% to 100%. As with PDLs, heterogeneity in inclusion criteria and laser parameters precluded meta‑analytic pooling; therefore, the results were synthesized narratively. These data indicate that Nd:YAG lasers can produce high clearance in selected cohorts, including reports of complete response in all treated lesions, but with considerable dispersion across studies. Reporting of adverse events varied, and events of bleeding or hemorrhagic bullae were documented in some series, aligning with the higher odds observed in the comparative analysis. The complete study-level data are presented in Table 2.

## Discussion

Across three head‑to‑head studies (n=224), complete‑clearance efficacy did not differ between PDLs and Nd:YAG lasers (OR, 1.29; 95% CI, 0.66–2.50; I²=0%). In contrast, hemorrhagic complications (hematoma/hemorrhagic bullae) occurred less frequently with PDLs (OR, 0.23; 95% CI, 0.07–0.74; I²=22%) [4–6]. Absolute event rates were approximately 3.5% for PDLs (4/115) versus 17.4% for Nd:YAG lasers (19/109), yielding an absolute risk difference of approximately 14% and an approximate number needed to harm of 7 when choosing Nd:YAG lasers over PDLs. Clinically, these results convey a pragmatic message that selection should be guided by safety and context rather than by expected clearance.

The strong hemoglobin absorption and short pulse durations of PDLs focus energy within superficial plexuses, whereas 1,064 nm Nd:YAG lasers penetrate more deeply with broader thermal effects [7]. Classic PDL histology demonstrates intravascular erythrocyte aggregation and thrombus formation, which is direct evidence for selective photothermolysis *in vivo* [8]. These differences offer a biologically plausible explanation for the lower hemorrhagic reactogenicity observed with PDLs in the direct comparison.

The broader laser literature has confirmed that both modalities are effective against warts [3]. Beyond laser‑versus‑laser comparisons, Nd:YAG lasers have demonstrated superiority to cryotherapy in a randomized controlled trial (RCT), underscoring the clinical efficacy of vascular‑targeted lasers [2]. Single-arm Nd:YAG series have demonstrated a wide range of outcomes (approximately 9%–100%), with higher‑fluence protocols frequently associated with hemorrhagic bullae and procedural pain [9-14]. In contrast, single‑arm PDL cohorts—especially those employing careful paring, targeting pinpoint bleeding, and using purpura as the endpoint at approximately 8 to 12 J/cm² with a 5-mm spot and short pulses—reveal consistent, often high, clearance with favorable tolerability [8,15-19]. These contextual data align with our head-to-head findings of efficacy parity, with a safety differential favoring PDLs.

For PDLs, effective parameters typically include fluence values of 6 to 15 J/cm², spot sizes of 5 to 7 mm, and pulse durations of 0.45 to 1.5 milliseconds, with purpura serving as the clinical endpoint. For Nd:YAG lasers, wider parameter ranges have been reported: fluence values of 100 to 300 J/cm², spot sizes of 2 to 7 mm, and pulse durations of 10 to 30 milliseconds. Higher fluence settings tend to improve clearance rates but increase hemorrhagic complications and procedural pain. The use of cooling devices (e.g., cold air or ice) appears to mitigate these adverse effects, although reports have been inconsistent across studies.

When cosmesis or minimizing bleeding risk is paramount (e.g., periungual or facial warts, or patient anticoagulant use), PDLs should be prioritized because of their lower hemorrhagic risk [4-6]. For thick, hyperkeratotic plantar lesions, Nd:YAG lasers remain a reasonable option, provided that the parameters (i.e., fluence, pulse duration, and spot size) and cooling are optimized, and patients are counseled regarding pain and hematoma risk [9-12]. When using an Nd:YAG laser, careful selection of anesthesia methods that consider the patient’s pain is necessary. For PDLs, standardizing paring and achieving a visible purpura endpoint improves consistency; limiting stacked pulses per zone on the sole may reduce hematoma formation while preserving ambulation [15,18].

Emerging energy-based and immunomodulatory approaches are reshaping the management strategies for recalcitrant cutaneous warts and complementing vascular-targeted lasers. In one RCT, combining Er:YAG ablation with long‑pulsed Nd:YAG treatment (1,064 nm) produced a higher single‑session complete response than Er:YAG ablation alone (48% vs. 29%), supporting a sequential “debulk‑then‑coagulate” strategy for thick, hyperkeratotic lesions [20]. In contrast, in palmoplantar disease, a comparative study found that adding 755 nm alexandrite laser to 1,064 nm Nd:YAG laser treatment did not significantly improve outcomes compared to Nd:YAG laser alone, suggesting a limited incremental value for routine dual-wavelength use [21].

The strengths of this study include a prespecified focus on refractory disease and reliance on direct comparisons for inferential conclusions, using single-arm data only for contextual interpretation. The limitations include the small number of comparative studies (k=3), mixed designs with nonrandomized components, and variability in adverse-event ascertainment.

In most settings, PDLs offer comparable clearance with fewer hemorrhagic events. Future trials should be adequately powered and stratified according to treatment site (periungual vs. plantar), lesion thickness, and histological features (e.g., parakeratosis, presence of koilocytes, vessel density, and epidermal thickness). Adopting patient-reported outcomes (e.g., pain, downtime, and successful cosmesis) and predefined safety endpoints with severity grading will further strengthen evidence for optimal laser selection.

## Figures and Tables

**Table 1.** Summary and comparison of studies using pulsed dye lasers (PDL) for refractory warts

Source	Study design; treatment	Quality rating	Laser parameter	Anesthetic approach	Treatment intervals (mean No. of treatments)	No. of patients (no. of warts)	Wart site/type	Resolution rate (%)	Adverse effect
Head-to-head study									
El-Mohamady et al. 2014 [6]	Intraindividual comparative (nonrandomized); PDL	2	PDL: 585 nm	EMLA 5% cream or 1 hr occlusion; lidocaine local injection for some patients treated by Nd:YAG	2 wk (5.05)	46	Plantar/ recalcitrant	73.9	Hematoma: 2.2%
			Spot size: 7 mm						Secondary bacterial infection: 4.3%
			Pulse duration: 0.5 ms						Persistent erythema: 2.2%
			Fluence: 8 J/cm²						Flare of foot eczema: 0%
			Cooling: none						
Shin et al. 2017 [5]	Retrospective comparative cohort; PDL	4	PDL: 595 nm	EMLA 5% cream without rubbing for 1 hour	2–4 wk (NS)	39	Mixed (periungual, common, plantar)/ recalcitrant	5.1	Hemorrhagic bullae: 2.56%
			Spot size: 7 mm		mean No. of treatment: 4.36				Posttreatment pain: 2.56%
			Pulse duration: 1.5 ms						Erythema: 5.13%
			Fluence: 10–14 J/cm²						Scarring: 0%
			Cooling: none						Dyspigmentation: 0%
									Ulceration: 0%
Ibrahim et al. 2021 [4]	Randomized intraindividual comparative clinical trial; PDL	1	PDL: 595 nm	Topical anesthesia in the form of a mixture of pridocaine and prilocaine cream for 1 hour	2 wk (4.97)	30	Plantar/recalcitrant	63.3	Hemorrhagic bullae: 6.6%
			Spot size: 7 mm						Secondary bacterial infection: 0%
			Pulse duration: 0.5 ms						Pain (Moderate to severe): 26.7%
			Fluence: 15 J/cm²						
			Cooling: none						
PDL single-arm study									
Tan et al. 1993 [8]	Prospective case series (single-arm); PDL	2	PDL: 585 nm	Not mentioned	1–3 (varied) wk (1.68)	39	Mixed (fingers and hands, plantar, periungual, other parts of body)/recalcitrant	71.8 (64% for fingers and hands; 50% for plantar; 86% for periungual; 83% for other parts of body)	NS
			Fluence: 6.25–7.5 J/cm²						
Webster et al. 1995 [16]	Retrospective case series (single-arm); PDL	4	PDL: 585 nm	No anesthesia	2–4 (varied) wk (2.4)	54	Mixed/recalcitrant	51.9 (71% for flat; 65% for palmar/plantar; 44% for common; 33% for periungual)	NS
			Fluence: 6.75–10 J/cm²						
Borovoy et al. 1996 [15]	Retrospective cohort (single-arm); PDL	4	PDL: 585 nm	Not mentioned	3 wk (2.38)	200	Plantar/recalcitrant	79.9	NS
			Spot size: 2–7 mm						
			Pulse duration: 0.45 ms						
			Fluence: 6–9 (plantar typically 6.5–7.25); average 8 J/cm²						
			Pulses: Up to 2 pulses per zone (‘double pulsing’) if purpura reaction is not noticed						
Jacobsen et al. 1997 [17]	Prospective case series (single-arm); PDL	2	PDL: 585 nm	Not mentioned	4–8 wk (1.84)	19 (122)	Mixed (periungual, plantar, face/neck, extremities)/recalcitrant	68.0	NS for recalcitrant wart
			Fluence: 8 J/cm²						
Schellhaas et al. 2008 [18]	Prospective case series (single-arm); PDL	2	PDL: 583–587 nm	No anesthesia	2 wk (3.7)	73 (366)	Mixed (hands or feet)/recalcitrant	89.0	Side effects and/or pain: 6.8%
			Spot size: 5 mm						
			Pulse duration: 0.45 ms						
			Fluence: 8–12 J/cm²						
			Pulses: 3–5 shots per site until livid discoloration						
Sethuraman et al. 2010 [19]	Retrospective cohort (single-arm); PDL	4	PDL: 585 nm	General: 33%	2.5–4 wk (3.1)	61, Children	Mixed (hands, combined extremities, plantar warts, combined face and extremities, perineal and perianal warts; face only)/recalcitrant	75.4 (93% for hands; 60% for combined extremities; 69% for plantar warts; 67% for combined face and extremities; 100% for perineal and perianal warts and face only	Hypopigmentation: 8%
			Spot size: 5 or 7 mm	EMLA: 49%					Hyperpigmentation: 2%
			Fluence: 6.5–9.5 (most at 7) J/cm²	Nerve block: 3%					Mild scarring and blistering: 2%
			Pulses: stacked pulses; average 3 per wart	EMLA & nerve block on separate occasions: 1%					Immediate itching after laser treatment: 3%
				General & EMLA 5% cream on separate occasions: 14%					

Quality rating scheme is modified from the Oxford Centre for Evidence-Based Medicine for ratings of individual studies: (1) properly powered and conducted randomized clinical trial, systematic review with meta-analysis; (2) well-designed controlled trial without randomization, prospective comparative cohort trial; (3) case-control studies, retrospective cohort study; (4) case series with or without intervention, cross-sectional study; (5) opinion of respected authorities, case reports. Nd:YAG, neodymium-doped yttrium aluminum garnet; EMLA, eutectic mixture of local anesthetics; NS, not specified.

**Table 2.** Summary and comparison of studies using neodymium-doped yttrium aluminum garnet lasers (Nd:YAG) laser for refractory warts

Source	Study design; treatment	Quality rating	Laser parameter	Anesthetic approach	Treatment intervals (mean No. of treatments for CR)	No. of patients (No. of warts)	Wart site/type	Resolution rate (%)	Adverse effect
Head-to-head study									
El-Mohamady et al. 2014 [6]	Intraindividual comparative (nonrandomized); Nd:YAG	2	Nd:YAG: 1,064 nm	EMLA 5% cream 1 hour occlusion; Lidocaine local injection for some patients treated by Nd:YAG	2 wk (4.65)	46	Plantar/recalcitrant	78.3	Hematoma: 28.3%
			Spot size: 7 mm						Secondary bacterial infection: 10.9%
			Pulse duration: 20 ms						Persistent erythema: 0%
			Fluence: 100 J/cm²						Flare of foot eczema: 4.3%
			Cooling: none						
Shin et al. 2017 [5]	Retrospective comparative cohort; Nd:YAG	4	Nd:YAG: 1,064 nm	EMLA 5% cream without rubbing for 1 hour	2–4 wk, NS (4.42)	33	Mixed (periungual, common, plantar)/recalcitrant	9.1	Hemorrhagic bullae: 6.06%
			Spot size: 5 mm						Post-treatment pain: 3.03%
			Pulse duration: 20 ms						Erythema: 0%
			Fluence: 240–300 J/cm²						Scarring: 0%
			Cooling: none						Dyspigmentation: 0%
									Ulceration: 0%
Ibrahim et al. 2021 [4]	Randomized intraindividual comparative clinical trial; Nd:YAG	1	Nd:YAG: 1,064 nm	Topical anesthesia in the form of a mixture of lidocaine and prilocaine cream for 1 hour	2 wk (4.63)	30	Plantar/recalcitrant	66.7	Hemorrhagic bullae: 13.3%
			Spot size: 5 mm						Secondary bacterial infection: 10%
			Pulse duration: 15 ms						Pain (moderate to severe): 100%
			Fluence: 100 J/cm²						
			Cooling: none						
Nd:YAG single-arm study									
Kimura et al. 2014 [11]	Single‑center prospective uncontrolled study; Nd:YAG	2	Nd:YAG: 1,064 nm	Ice for 18 patients; 1% lidocaine injection for 6 out of 18 ice-applied patients	4 wk (NS)	20 (34)	Mixed (palmoplantar, fingers, periungual/subungual, toes)/recalcitrant	55.88 (39 for plantar area; 73 for Fingers; 100 for paronychial/subungual areas; 50 for toes)	Internal hemorrhages and blood-filled blisters for most of the treated lesions
			Spot size: 5 mm						
			Pulse duration: 15 ms						
			Fluence: 150–185 J/cm²						
			Cooling: cold air & ice						
Smith et al. 2015 [10]	Retrospective postal questionnaire audit; Nd:YAG	4	Nd:YAG: 1,064 nm	0.5% plain lidocaine solution local injection for 17 patients	1–4 (varied) wk (3.6; 2.5 for single verrucas on one foot; 4.0 for multiple verrucas on one foot; 3.7 for bilateral verrucas)	53	Plantar/recalcitrant	69.81 (81.8 for unilateral single verrucas; 68.1 for unilateral multiple; 65 for bilateral)	NS
			Spot size: 2 mm						
			Pulse duration: 25 ms						
			Fluence: 240 J/cm²						
			Cooling: cold air (Zimmer Cryo 6)						
Bingol et al. 2015 [9]	Retrospective case series; Nd:YAG	4	Nd:YAG: 1,064 nm	Topical anesthetics and/or local lidocaine injection	NS (1.116)	51 (146)	Hand/recalcitrant	100.0	Thin scar: 11.64%
			Spot size: 3 mm						Hyperpigmentation: 5.48%
			Pulse duration: 23 ms						Hypopigmentation: 0%
			Fluence: 180–200 J/cm²						
			Cooling: cold air (Cryo 6)						
Ghonemy 2017 [12]	Comparative study; quasi‑randomized by odd/even assignment; Nd:YAG	1	Nd:YAG: 1,064 nm	EMLA 5% cream	4 wk (4.125)	15 (71)	Plantar/recalcitrant	53.33	Pain: 40.0%
			Spot size: 5 mm						Bulla: 46.7%
			Pulse duration: 10 ms						Hemorrhagic bulla: 0%
			Fluence: 120 J/cm²						
			Cooling: none						
Sanad et al. 2018 [14]	Randomized (method not described) two‑arm comparative trial; Nd:YAG	1	Nd:YAG: 1,064 nm	Mepivacaine hydrochloride local injection	4 wk (1.6)	10	Mixed (common, plantar)/recalcitrant	100.0	Secondary infection: 0%
			Spot size: 5 mm						Scarring: 20%
			Pulse duration: 30 ms						Hypopigmentation: 10%
			Fluence: 200 J/cm²						Hyperpigmentation: 10%
			Cooling: none						Bleeding: 50%
									Hemorrhagic bullae: 50%
									Nail dystrophy: 10%
Khattab and Khashaba 2020 [13]	Randomized lesion‑level split‑body trial (lesions allocated left/right halves); Nd:YAG	1	Nd:YAG: 1,064 nm	EMLA 5% cream; ice pack application	4 wk (3.1)	38 (132)	Mixed (palmoplantar, arm, face, chin, chest)/recalcitrant	69.7	Blisters: 9.09%
			Spot size: 5 mm						Crusts: 18.18%
			Pulse duration: 10 ms						Eschars: 6.06%
			Fluence: 120 J/cm²						Hyperpigmentation: 6.06%
			Cooling: ice‑pack post‑procedure						Pain (moderate to severe: 33.33%

Quality rating scheme is modified from the Oxford Centre for Evidence-Based Medicine for ratings of individual studies: (1) properly powered and conducted randomized clinical trial, systematic review with meta-analysis; (2) well-designed controlled trial without randomization, prospective comparative cohort trial; (3) case-control studies, retrospective cohort study; (4) case series with or without intervention, cross-sectional study; (5) opinion of respected authorities, case reports. EMLA, eutectic mixture of local anesthetics; NS, not specified.
